# Insights into organ-specific pathogen defense responses in plants: RNA-seq analysis of potato tuber-*Phytophthora infestans* interactions

**DOI:** 10.1186/1471-2164-14-340

**Published:** 2013-05-23

**Authors:** Liangliang Gao, Zheng Jin Tu, Benjamin P Millett, James M Bradeen

**Affiliations:** 1 Department of Plant Pathology, University of Minnesota, Saint Paul, MN, 55108, USA; 2 Biomedical Statistics and Informatics Division, Mayo Clinic, Rochester, MN, 55905, USA

**Keywords:** *Solanum tuberosum*, Next-generation sequencing (NGS), RNA-seq, *Phytophthora infestans*, FPKM (fragments per kilobase of exon per million mapped reads), JA (jasmonic acid), SA (salicylic acid), ET (Ethylene), qRT-PCR

## Abstract

**Background:**

The late blight pathogen *Phytophthora infestans* can attack both potato foliage and tubers. Although interaction transcriptome dynamics between potato foliage and various pathogens have been reported, no transcriptome study has focused specifically upon how potato tubers respond to pathogen infection. When inoculated with *P. infestans*, tubers of nontransformed ‘Russet Burbank’ (WT) potato develop late blight disease while those of transgenic ‘Russet Burbank’ line SP2211 (+*RB*), which expresses the potato late blight resistance gene *RB* (*Rpi-blb1*), do not. We compared transcriptome responses to *P. infestans* inoculation in tubers of these two lines.

**Results:**

We demonstrated the practicality of RNA-seq to study tetraploid potato and present the first RNA-seq study of potato tuber diseases. A total of 483 million paired end Illumina RNA-seq reads were generated, representing the transcription of around 30,000 potato genes. Differentially expressed genes, gene groups and ontology bins that exhibited differences between the WT and *+RB* lines were identified. *P. infestans* transcripts, including those of known effectors, were also identified.

**Conclusion:**

Faster and stronger activation of defense related genes, gene groups and ontology bins correlate with successful tuber resistance against *P. infestans*. Our results suggest that the hypersensitive response is likely a general form of resistance against the hemibiotrophic *P. infestans*—even in potato tubers, organs that develop below ground.

## Background

Cultivated potato is the world’s third most important human food crop and the number one non-grain food commodity (FAOSTAT 2010). Unfortunately, potato is also host of a broad range of pathogens [1]. Late blight disease is caused by *Phytophthora infestans*, resulted in the Irish Potato Famine in the 1840s, and still today results in multi-billion dollar losses worldwide annually. *P. infestans* is a notorious plant destroyer with the capacity to attack both potato foliage and tubers. Foliage resistance against late blight does not guarantee tuber resistance—contrasting disease resistance phenotypes can be evident in comparing foliage and tubers from a single genotype [2].

Gene *RB* (*Rpi-blb1*) [3,4], a disease resistance gene cloned from a wild potato species, confers broad-spectrum foliar resistance against all major late blight pathogen isolates. The gene has been introduced into several potato cultivars using transgenic approaches [5]. Transgenic lines have been or are currently being tested for eventual commercial release in Europe, India, Bangladesh, US and other places. Halterman et al. [6] reported that the four foliar late blight resistant +*RB* transgenic potato lines examined in their study lacked statistically significant tuber blight resistance. However, in a more extensive survey of *+RB* transgenic potato lines, we identified two transgenic lines with unusually high *RB* transcript levels that were late blight resistant in both foliage and tuber [6]. Thus, *RB* has the potential to function in the tuber and the *RB*-potato tuber-*P. infestans* interaction provides a tractable system to study how potato tubers defend against plant pathogens.

Next-generation sequencing (NGS) technologies are fast evolving and are transforming biology research [7,8]. Genome sequences of potato and *P. infestans* have been published [9,10], making sequencing-based transcriptome studies (RNA-seq) more accessible to potato late blight researchers. RNA-seq is a relatively new approach towards study of the transcriptome [11,12]. To our knowledge, there has been no RNA-seq study focused on potato-microbe interactions. Indeed, one NGS-based transcriptome study focused on the interaction between potato and *P. infestans* has been published [13]. However, that study utilized the DeepSAGE method, not RNA-seq. DeepSAGE differs substantially from RNA-seq and researchers in that study relied heavily on assembled tags; the newly available genome sequence data were mostly not utilized. Furthermore, that and all other published studies focused on potato foliage transcriptome dynamics [13-15]; no previous study has reported transcriptome dynamics of potato tubers in response to pathogen attack.

In this study, we employed RNA-seq to study the transcriptome dynamics of potato tuber- *P. infestans* interactions in compatible and incompatible potato genotypes*.* We employed genome-wide sequence data from both potato and *P. infestans* in our analyses. Differentially expressed genes and ontology bins were identified that distinguish compatible and incompatible interactions. Transcripts of *P. infestans*, including those from candidate effectors, were also identified. Our study has important implications for potato *R* gene deployment and contributes to scientific understanding of organ-specific defense regulation in plants.

## Results

### The +*RB* transgenic line exhibits enhanced tuber late blight resistance

**Figure 1:**
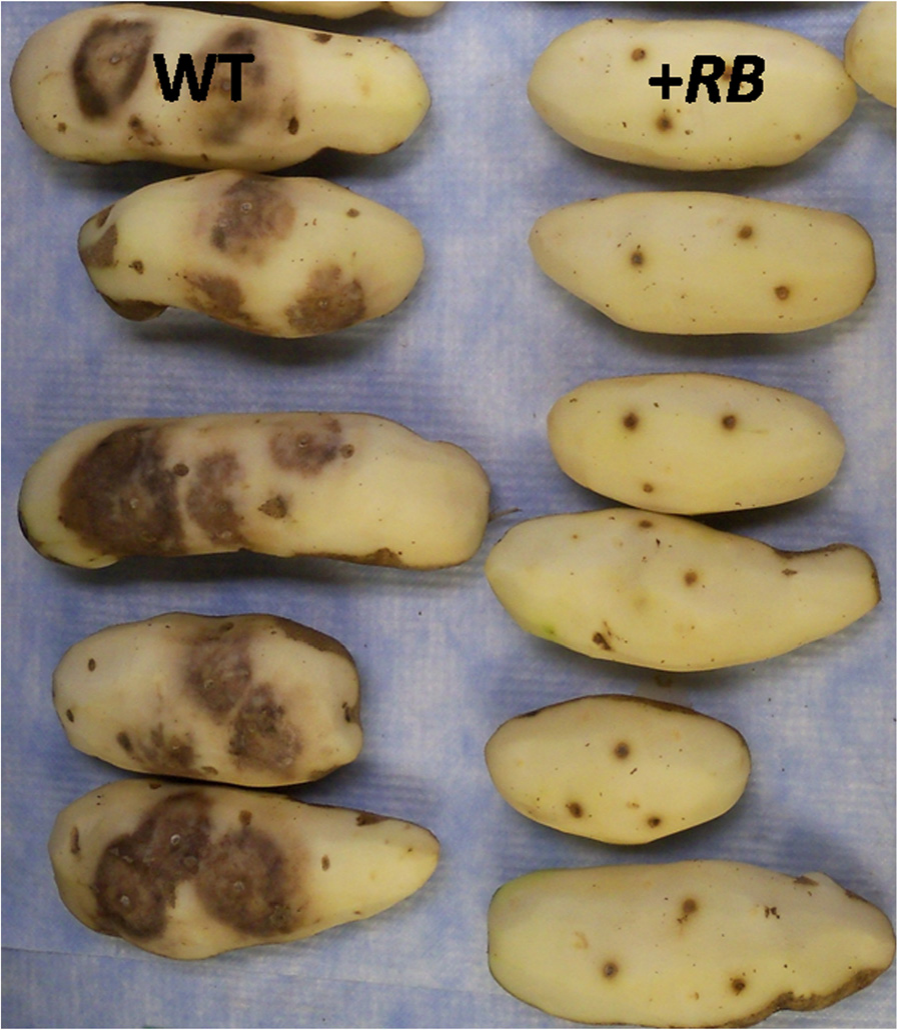
**Gene *RB *confers enhanced tuber late blight disease resistance*.*** Field grown tubers of nontransformed ‘Russet Burbank’ (WT) and SP2211 (*+RB*), a transformed ‘Russet Burbank’ line carrying the *RB* transgene, were mechanically wounded, inoculated with *Phytophthora infestans,* and incubated for 11 days under conditions that favor disease development. Tubers were peeled to allow assessment of diseased tissues. WT tubers consistently show robust tuber late blight disease development, revealed as darkened tissue radiating from inoculation sites. In contrast, *+RB* tubers displayed no tuber late blight disease. Note that brown spots present on *+RB* tubers are in response to mechanical wounding, not late blight disease; similar wound response was observed in water-inoculated tubers (not shown).

In previous field-based evaluations, the +*RB* line examined in this study, SP2211, was ranked as the third most foliar late blight resistant of 57 transgenic lines tested and was rated as “Resistant” to foliar late blight [5]. In contrast, the WT line (nontransformed ‘Russet Burbank’) was rated as “Susceptible” to foliar late blight [5]. Here, in replicated whole tuber assays performed six weeks after harvest, the +*RB* line showed no tuber late blight disease after normalization to the water-inoculated controls, whereas the WT line showed clear tuber late blight disease development (Figure 1). Results from two sets of whole tuber experiments were in agreement (32 of 33 *P. infestans* inoculations of WT tubers resulted in disease, none of the 60 inoculation sites in +*RB* tubers developed late blight disease). Thus, while WT is susceptible to *P. infestans* infection in both foliage and tuber, the *RB* transgene renders the +*RB* line SP2211 resistant to *P. infestans* infection in both foliage and tuber. *RB* gene transcription in the tubers of the *+RB* line was unaltered by inoculation with *P. infestans* at 0, 24, and 48 hpi (p>0.05).

### RNA-seq reads aligned well with the potato reference genome sequence

**Figure 2:**
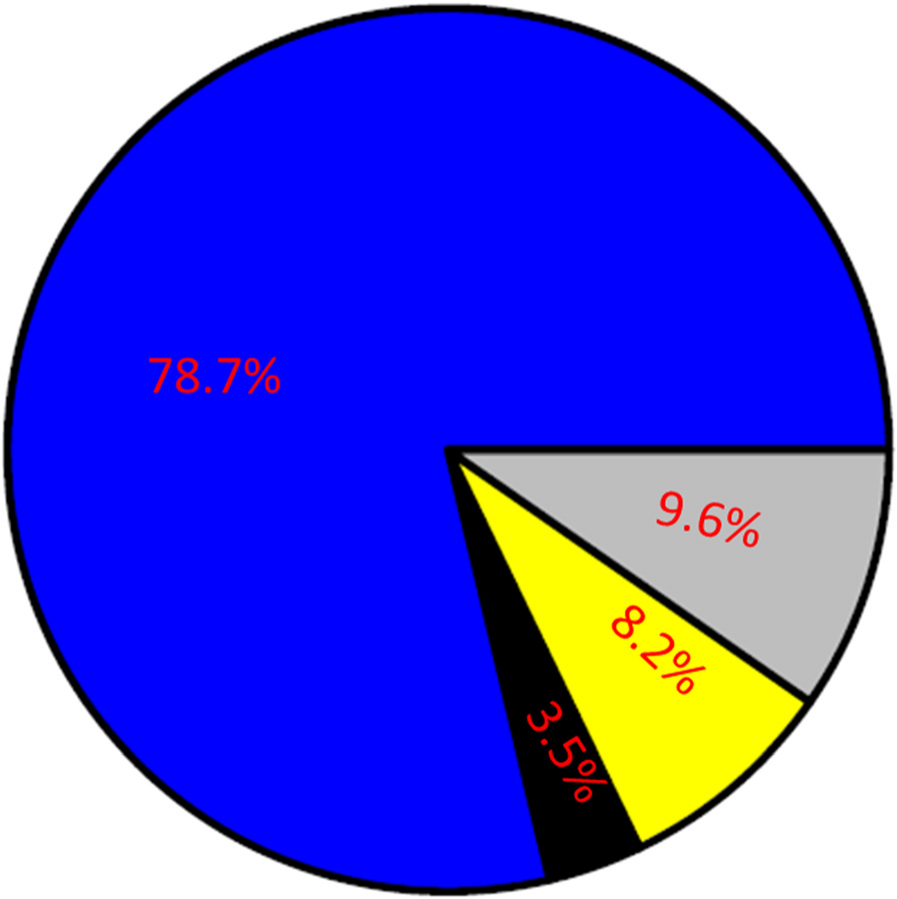
**A majority of RNA-seq reads map uniquely to the potato reference genome sequence.** A pie chart summarizing results of alignment of Illumina RNA-seq reads from 36 water- or *P. infestans-*inoculated WT and *+RB* tuber samples to the potato reference genome sequence. The grey portion of the chart represents RNA-seq reads that failed quality checks and were filtered out of our data set (9.6% of all RNA-seq reads). The remaining 90.4% of RNA-seq reads passed quality checks. The blue portion of the chart represents reads that mapped uniquely to the potato genome sequence (78.7% of all RNA-seq reads). The black portion of the chart represents reads that mapped to multiple locations of the potato genome sequence (3.5%). The yellow portion of the chart represents reads that failed to map to the potato genome sequence (8.2%), including <0.01% of RNA-seq reads that mapped to *P. infestans* transcripts.

A total of 36 RNA samples, collected from three biological replicates of *P. infestans-* or water-inoculated tuber tissues of two potato genotypes (WT and +*RB*) at three time points post inoculation, were subjected to RNA-seq. Approximately 483 million paired end reads were generated, yielding an average of 13.4 million paired end reads per sample. A total of 436.3 million Illumina reads (90.3%) passed quality filtering. The majority of reads (380.2 million or 78.7% of all reads) could be mapped uniquely to one location within the doubled monoploid (DM) potato reference genome sequence [9] (Figure 2). An additional 16.6 million reads (3.5%) were mapped to multiple locations within the reference genome sequence (Figure 2).

We detected transcription of 29,319 potato genes based on cufflinks FPKM (fragments per kilobase of exon per million mapped reads) information and gene models reported by the Potato Genome Sequencing Consortium (PGSC) [9]. Wang et al. [16] demonstrated that a data set of 10 million RNA-seq reads represented about 80% of annotated chicken genes, concluding that RNA-seq is a viable alternative to the microarray for transcriptome study. In this study, we detected more than 75% of PGSC potato gene models—a percentage similar to that reported by Wang et al. [17]. This scale of gene detection is higher than reported for any previous potato-*P. infestans* transcriptome study. Importantly, 19.8% of RNA-seq reads that passed quality filters were mapped to regions outside of the current potato reference genome gene models (or predicted exons), suggesting further refinement of gene annotations associated with the potato reference genome sequence may be warranted. Nonetheless, qRT-PCR results correlated well with RNA-seq data with an average Pearson correlation coefficient of r=0.86 (Additional file 1). Together, our results suggest that RNA-seq and qPCR approaches can be cross-validated, confirming that current potato gene models [9] are appropriate for functional genomics studies of potato tubers.

### Overall transcriptome dynamics

**Figure 3:**
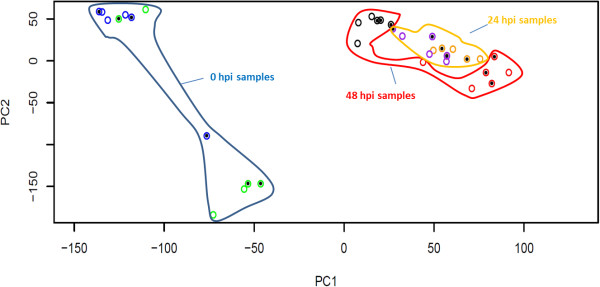
**Time and treatment have larger influences than genotype on overall transcriptome differences.** FPKM (Fragment per kilobase of exon per million mapped reads) values for transcriptome sets from 36 potato tuber samples were subjected to PCA using the R statistical package [53]. Blue circles represent *P. infestans*-inoculated tuber samples collected at 0 hpi; green circles represent water-inoculated samples at 0 hpi; orange circles represent *P. infestans*-inoculated samples collected at 24 hpi; purple circles represent water-inoculated samples collected at 24 hpi; red circles represent *P. infestans*-inoculated samples collected at 48 hpi; black circles represent water-inoculated samples collected at 48 hpi. Circles containing dots were collected from the transgenic line SP2211 (*+RB*); circles without dots were collected from nontransformed ‘Russet Burbank’ (WT). Note that tuber samples collected at a similar time tend to cluster, regardless of genotype of origin.

A principal component analysis (PCA) of log2 transformed FPKM values for 29,319 genes from the 36 RNA-seq samples is shown in Figure 3. Together, PC1 and PC2 explained >40% of the total variance of this dataset. Samples collected at 0 hpi were distinct from all other samples, with PC1 providing clear separation of 0 hpi samples from 24 hpi and 48 hpi samples. PC1 also differentiates water- and *P. infestans*-inoculated samples at 48 hpi. Overall, the distribution of +*RB* samples is similar to that of corresponding WT samples. These results indicate that time and treatments (*P. infestans-* vs. water-inoculation) play a greater role than genotype in defining overall transcriptome dynamics. This results is expected given that the *+RB* line was created by transformation of the WT line with the *RB* transgene.

### Differentially expressed (DE) genes

**Figure 4:**
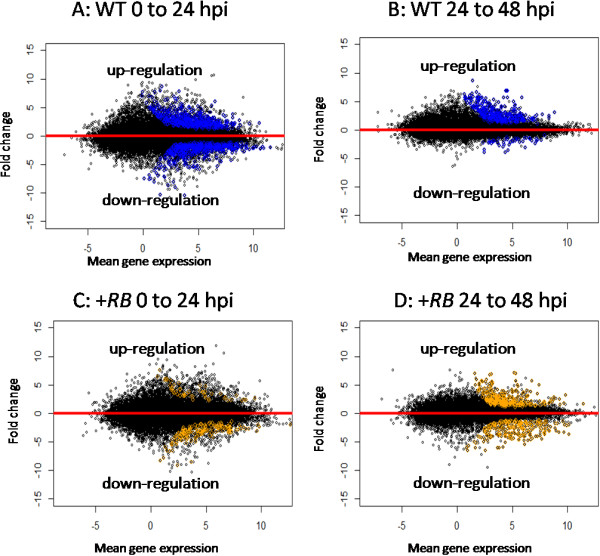
**Between time point transcriptome dynamics reveal different patterns of gene regulation in compatible and incompatible potato tuber – *Phytophthora infestans *interactions.** MA plots (hybridization intensity plotted against the fold change in expression) displaying between time point transcriptome dynamics in nontransformed ‘Russet Burbank’ (WT) and transgenic SP2211 (+*RB*) lines in response to *P. infestans*. X-axes indicate mean gene expression levels [0.5*(log2(FPKM1)+log2(FPKM2))] across the two selected time points for each comparison. Y-axes indicate fold change values [log2(FPKM1/FPKM2), where FPKM1 represents the later time point and FPKM2 represents the earlier time point] across the selected time points. A: changes in WT transcriptome dynamics from 0 to 24 hpi; B: changes in WT transcriptome dynamics from 24 hpi to 48 hpi; C: changes in +*RB* transcriptome dynamics from 0 hpi to 24 hpi; D: changes in *+RB* transcriptome dynamics from 24 hpi to 48 hpi. Within each panel, colored dots represent genes that are significantly (FDR<0.001) differentially regulated among comparisons. Black dots represent genes that are not significantly differentially regulated. Note that the WT line shows mostly up-regulation of genes during later stages of infection (panel B) while the *+RB* line displays approximately equal up- and down-regulation of genes (panel D).

We also identified 2,531 genes that are differentially expressed (DE) during at least one of the between time transitions in *P. infestans*-inoculated samples (Additional file 2). Transcriptome dynamics of both genotypes in *P. infestans-* compared to water-inoculated samples during transitions from 0 hpi to 24 hpi and from 24 hpi to 48 hpi were visualized using MA plots (hybridization intensity plotted against the fold change in expression; Figure 4). Interestingly, at later stages of infection (24 hpi – 48 hpi), WT tubers exhibited mostly up-regulation of genes, whereas the +*RB* line displayed approximately equal numbers of up- and down-regulated genes (Figure 4), potentially reflecting contrasting rates of pathogen proliferation (as indicated by total pathogen reads counts, see results sections below) and corresponding host response patterns.

Transcriptome dynamics of between line comparisons were also visualized using MA plots (Additional file 3). Consistent with PCA which shows that genotype has a lesser role (compared to time or treatment) in defining overall transcriptome dynamics, fewer genes were determined to be DE during between line comparisons, despite a trend of increasing numbers of DE genes over time (Additional file 3). This suggests that both the WT and the *+RB* line use that same set of genes to respond to *P. infestans* attack but that there are significant differences in the temporal regulation of these genes.

Next we employed hierarchical clustering to visualize the DE genes identified through between time point comparisons. Despite a preponderance of shared DE gene regulation patterns, groups of genes that distinguish compatible and incompatible interactions were still identified (Additional files 3 and 4). These include WRKY (PGSC0003DMG400008188) and ethylene response (PGSC0003DMG400025989, *ERF1*) transcription factors. The *ERF1* transcription factor gene is a likely key element in the integration of JA (jasmonic acid) and ET (ethylene) signals for the regulation of defense responses [17].

### The +*RB* transgenic line has faster and stronger induction of DE genes

**Figure 5:**
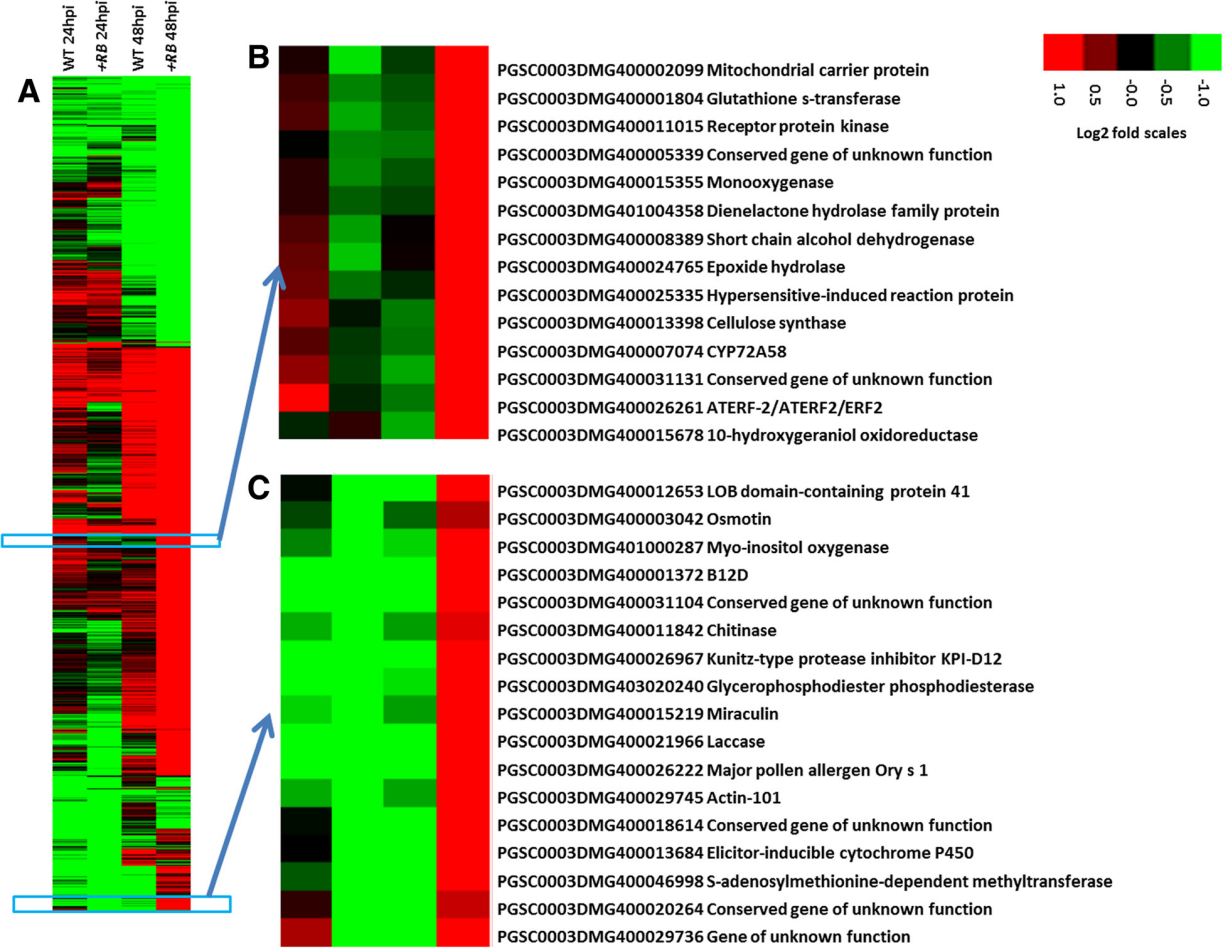
**Hierarchical clustering of differentially expressed (DE) genes in potato tubers following inoculation with *Phytophthora infestans.*** Tubers of nontransformed ‘Russet Burbank’ (WT) and transgenic SP2211 (*+RB*) were inoculated with *P. infestans* or water*.* Tuber samples collected 0, 24, and 48 hpi were subjected to RNA-seq, revealing a total of 1,102 DE genes between water- and *P. infestans-*inoculated comparisons within the same genotype and the same time. Log2(FPKM_p.inf/FPKM_mock) values were used to cluster 1,102 DE genes (FDR<0.001) in Cluster 3.0 [51] using uncentered correlation and the complete linkage method. Results were visualized using Treeview [51]. (**A**) Global visualization of the 1,102 DE genes; (**B**) A small gene cluster differentially regulated in +*RB* and WT at 24 and 48 hpi; (**C**) A small gene cluster differentially regulated in +*RB* and WT only at 48 hpi. Red indicates genes that are up-regulated, green indicates genes that are down-regulated.

**Figure 6:**
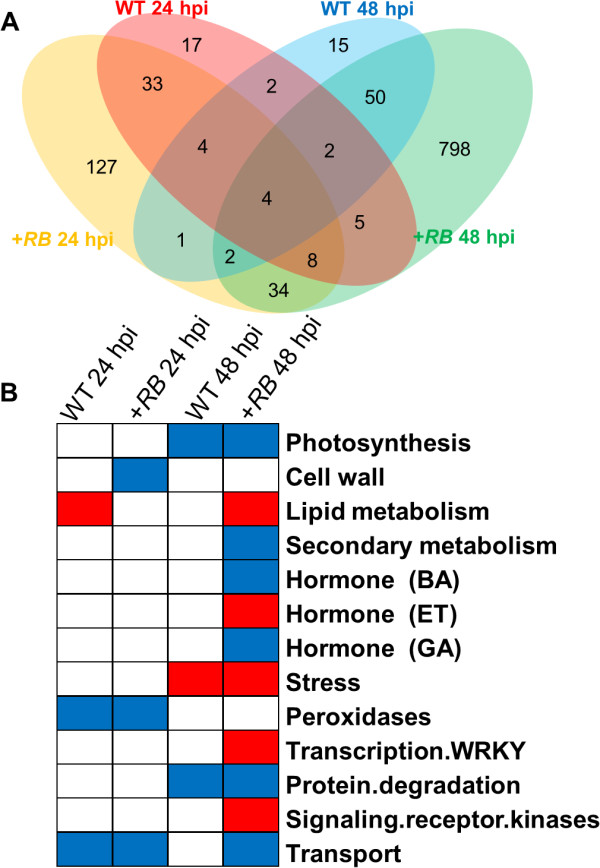
**Tubers of the +*RB *line have a higher frequency of differentially expressed (DE) genes 48 hours post inoculation (hpi) with *Phytophthora infestans, *compared to WT (24 and 48 hpi) and *+RB *at 24 hpi.** Tuber samples collected 0, 24, and 48 hpi were subjected to RNA-seq, revealing a total of 1,102 DE genes between water- and *P. infestans-*inoculated comparisons within the same genotype and the same time. (**A**) All 1,102 DE genes were analyzed using the “Venn count” function in the limma packages of R [53] and results were summarized as a Venn diagram. Red: WT 24 hpi; yellow: +*RB* 24 hpi; blue: WT 48 hpi; green: +*RB* 48 hpi. The results show that the *+RB* line is the main contributor of DE genes during water- vs. *P. infestans*-inoculated comparisons. (**B**) All 1,102 DE genes were also assigned to a MapMan ontology based on the Mercator mapping file (see methods), and subjected to Fisher’s exact test. Bins in red were significantly up-regulated; bins in blue were significantly down-regulated; transcription of bins in white did not change significantly. The results indicate that ontology bins encompassing ET metabolism and signaling are enriched for DE genes in *+RB* but not in WT at 48 hpi.

A total of 1,102 DE genes were identified when water- and *P. infestans*-inoculated samples were compared (between treatment comparisons) at the same time points (24 hpi or 48 hpi). Of these, 959 were DE only in the *+RB* line, 34 were DE only in the WT line, and 109 were DE in both lines. We identified two representative hierarchical clusters, comprising a total of 31 genes, showing interesting regulation patterns at 48 hpi (Figure 5). In WT, these 31 genes were suppressed (compared to water-inoculated); in +*RB*, these 31 genes were induced (compared to water-inoculated). These genes include ethylene response factors (*ERF2*), cellulose synthase, chitinase, and elicitor inducible cytochrome P450. Two genes specifically associated with the hypersensitive response (HR), PGSC0003DMG400025335 [*Hypersensitive-induced reaction protein* (*HIR*); Figure 5] and PGSC0003DMG400027473 (*Hypersensitive response assisting protein*) were significantly induced in the +*RB* line but not in the WT line at 48 hpi. It is important to highlight that the +*RB* line displayed 903 DE genes at 48 hpi, accounting for about 82% of the total DE genes identified during between treatment comparisons (Figure 6). These results suggest that the +*RB* line responds to *P. infestans* attack by rapid differential regulation of large sets of genes, whereas the WT line is slower to respond (e.g., 48 hpi).

### Ontology bins and gene groups distinguishing compatible and incompatible interactions

Mapman ontology enrichment analysis of DE genes (Figure 6) revealed that WRKY transcription factors are up-regulated in +*RB* but not in WT at 48 hpi. Strikingly, 13 of the 14 DE (during water- vs. *P. infestans-*inoculated comparisons) WRKY transcription factor genes show significant induction in the +*RB* line at 48 hpi (Additional file 5). In contrast, none of the 14 WRKY transcription factor genes showed statistically significant (FDR<0.001) induction in the WT line at 48 hpi. Homologs of several of these WRKY transcription factors have been previously reported to be involved in defense against *Phytophthora* spp. and other pathogens [18,19]. We hypothesize that DE WRKY transcription factors identified in this study are downstream regulatory components of *RB*-mediated defenses in potato tubers.

**Figure 7:**
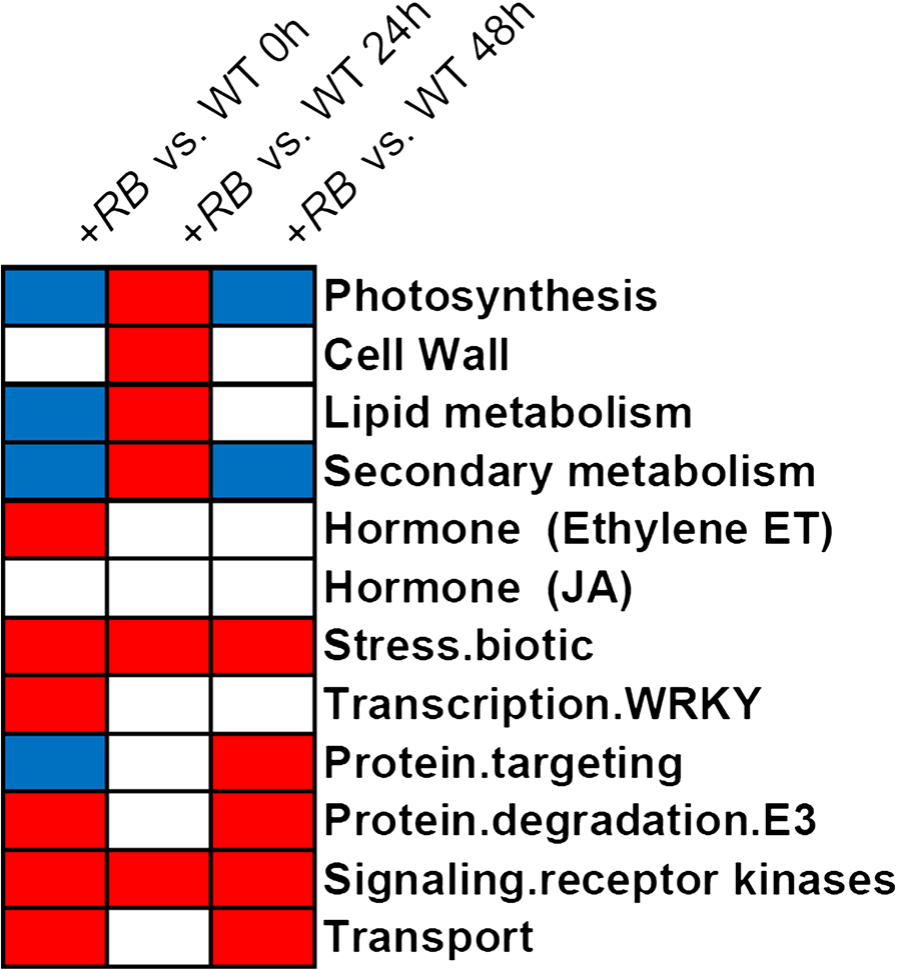
**Stronger activation of defense related genes or gene groups correlates with successful tuber resistance against *P. infestans*.** Tubers of ‘Russet Burbank’ (WT) and SP2211 (*+RB*) were inoculated with *P. infestans* and water. We compared the RNA-seq FPKM counts for WT and +*RB* using all 39,031 gene models included in the Potato Genome Sequencing Consortium (PGSC) v3 dataset (i.e., all genes were included, regardless of whether or not a given gene was DE). Genes were grouped into ontology bins using a Mapman mapping file. Each column represents a comparison between the two genotypes at a defined time point post inoculation, as indicated. Bins in blue are transcribed at higher levels in WT than in *+RB;* bins in red are transcribed at higher levels in *+RB* than in WT; bins in white did not significantly differ in transcript levels between WT and *+RB.* Results indicate that faster and stronger activation of defense bins, most notably biotic stress response and receptor kinase bins, occurred in tubers of the tuber late blight resistant +*RB* line.

Very importantly, we discovered that when WT and +*RB* were compared directly, the +*RB* line displays stronger transcription of genes within receptor kinase and defense related bins, even at 0 hpi (Figure 7 and Additional file 6). It is especially relevant to note that many of the bins commonly associated with plant defense, including receptor kinases and *PR* (pathogenesis-related) protein genes are more highly expressed in the +*RB* line. Interestingly, WT actually has much higher transcription of similar defense-related bins at 24 hpi (but not 48 hpi) if an indirect comparison (adjusting FPKM values of *P. infestans*-inoculated samples by FPKM values of water-inoculated samples of the same genotype) was made, indicating that WT and +*RB* probably share large sets of defense components. Thus, WT responds to *P. infestans* by up-regulation of sets of defense response genes that are constitutively transcribed at higher levels in the *+RB* line even in the absence of the pathogen (i.e., 0 hpi). But slower activation of defense related genes in WT is insufficient to prevent disease development, resulting in a tuber blight susceptible phenotype. These results were corroborated by an independent pilot RNA-seq study (Illumina GAIIx single end reads) using field grown tubers of the same genotypes from a different year (Additional file 7). Collectively, these findings suggest that faster and stronger expression of defense related genes plays a role in enhanced disease resistance in the *+RB* line.

### RNA-seq reads mapping to *Phytophthora infestans* reference transcripts

A total of 17,353 paired end sequence reads were mapped to the *P. infestans* reference transcript set [10], representing the transcription of over 4,600 genes. Ninety-six *P. infestans* transcripts are predicted to encode RxLR effectors [10].

Throughout the infection time course, a trend of increasing mappable *P. infestans* reads is evident for WT samples. This increase of mappable reads was not seen in the +*RB* line from 24 hpi to 48 hpi (Additional file 8) and a majority (>82%) of all mapped reads originated from infected WT potato tuber samples collected at 48 hpi. This is in agreement with tuber late blight disease development observed in WT but not *+RB* tubers. In total, a majority of all *P. infestans* reads (>95%) were from infected WT potato tuber samples; less than 5% of the reads were from infected *+RB* potato tuber samples (Additional file 9).

Class I and II ipiO proteins, members of the RxLR family of putative effectors, induce avirulence responses in potato lines containing *RB*[20-22]. After inoculation (24, 48 hpi), class I *ipiO* transcripts were detected (though in low abundance) in five of six tuber samples collected from the *P. infestans*-inoculated WT line but from no samples derived from the +*RB* line (Additional file 10). This is probably due to suppression of *P. infestans* proliferation in the +*RB* line.

## Discussion

### Faster and stronger activation of defense related genes, gene groups and ontology bins correlates with successful tuber defense

Direct comparison between WT and *+RB* lines at 0 hpi revealed a total of 11 DE genes (Additional file 3). Seven of the eleven DE genes were more highly transcribed in +*RB*. These include Carbonic anhydrase (PGSC0003DMG400006956), Aquaglyceroporin (PGSC0003DMG400009604), Cytochrome P450 (PGSC0003DMG400015185), Malic enzyme (PGSC0003DMG401026923), and 1-aminocyclopropane-1-carboxylate oxidase (ACO) (PGSC0003DMG401026923). Interestingly, these genes or homologs of these genes were previously reported to be involved in basal or pathogen-induced defense responses in plants [14,23,24]. The four remaining DE genes [protoporphyrinogen oxidase (PGSC0003DMG400001117), phenylcoumaran benzylic ether reductase (PGSC0003DMG400003691), DNA binding protein (PGSC0003DMG400003902), Major Latex (PGSC0003DMG400008811)] are transcribed at higher levels in WT than in the *+RB* line. Each of these genes has also been associated with plant defense [25-28]. Together, our results demonstrate that the *+RB* line differs from WT in the constitutive transcriptional regulation of defense related genes.

GO enrichment analysis of DE genes identified during between treatment comparisons (Figure 6) suggests that +*RB* has faster response to pathogen attack, as evidenced by higher and statistically significant induction of ontology bins commonly associated with plant defense (e.g., ET, WRKY, Signaling receptor kinases) at 48 hpi. One notable example is WRKY transcription factors (Additional file 5), with 13 out of 14 WRKY transcription factors significantly induced in +*RB.* These same transcription factors are also induced, but at nonsignificant levels, in the WT line, documenting that more potent and rapid activation of defense related genes, rather than novel resistance responses *per se,* might play a role in enhanced tuber resistance in the +*RB* line.

Furthermore, when a more holistic approach (using all available data points: 39,031 gene transcript values per sample) was employed to identify differentially regulated ontology bins, various defense related ontology bins were discovered (Figure 7). *+RB* potato tubers displayed faster and stronger transcription of genes within bins commonly associated with plant defense such as receptor kinases and pathogenesis-related (*PR*) genes (Figure 7). Together, these results suggest that the *+RB* line is tuber late blight resistant due to faster and stronger activation of defense related components*.*

Cao et al. [29] found that increased transcription of the rice *Xa3* resistance gene correlated with enhanced expression of defense-responsive genes and an enlarged resistance spectrum to *Xanthomonas oryzae* pv. *oryzae* (Xoo). Previously we demonstrated that higher transgene *RB* copy numbers correlated with both higher *RB* transcript levels and enhanced late blight resistance in the foliage [5]. The *+RB* line employed in this study, SP2211, displayed the highest tuber transgene transcription levels among 11 transgenic lines examined [6]. Thus, the unusually high levels of *RB* gene transcription in the tubers of SP2211 correlates with faster and stronger activation of defense related components (Figures 6, 7). The faster and stronger up-regulation of these defense-related components very likely contributed to the observed successful defense against *P. infestans* (Figure 1) in this line. One interesting observation is that WT actually has much higher transcription of similar defense-related bins at 24 hpi (but not 48 hpi) in indirect (adjusting FPKM values of *P. infestans*-inoculated samples by FPKM values of water-inoculated samples of the same genotype) comparisons. Collectively, our data support a model of tuber blight resistance resulting as a function of faster and stronger expression of defense related genes due to high *RB* transcript levels.

### HR is likely a general form of resistance to *P. infestans*, even in potato tubers

Prevailing scientific thought suggests that the phyllosphere frequently encounters biotrophic or hemibiotrophic pathogens, against which the HR is effective. In contrast, roots are more likely to encounter necrotrophic microbes that would theoretically benefit from the HR [30]. Consistently, working in Arabidopsis, Hermanns et al. [31] showed that HR is present in above ground incompatible host-pathogen interactions, but absent during the same host-pathogen interaction in the roots—despite R gene transcription in both leaves and roots. These authors concluded that R gene function is modulated in an organ-specific but mechanistically poorly defined manner for the purpose of suppressing HR in the roots. Observations of differential late blight resistance levels in potato foliage and tubers within a single genotype [2] suggest that R gene function in potato may also be modulated in an organ-specific manner. But the potato tuber, although it develops below ground, is a modified stem, not a root. Previous studies [32,33] have suggested that HR plays a role in tuber defense. The identification of SP2211, a *+RB* transgenic line with both foliar and tuber late blight resistance provided opportunity to examine the role of HR in defending non-root organs against pathogen attack.

*P. infestans* is a hemibiotrophic pathogen and Kamoun et al. [34] argued that HR is likely a general form of resistance against *Phytophthora* pathogens. In agreement, Chen and Halterman [35] demonstrated that *RB* triggers an HR in potato foliage challenged with *P. infestans.* In examining *RB-*mediated tuber responses to *P. infestans*, we identified DE genes associated with the HR (potato *HIR* and hypersensitive response assisting genes) that are highly up-regulated in pathogen-inoculated tuber samples of SP2211 compared to water-inoculated tuber samples at 48 hpi. Previous studies have shown that the pepper homolog of *HIR* is a positive regulator of hypersensitive cell death [36,37]. Qi et al. [38] also demonstrated that the NBS-LRR protein RPS2 forms complexes with AtHIR proteins in Arabidopsis and tobacco plants, providing mechanistic insight into R protein function and the HR. We hypothesize that RB and HIR might form similar complexes in potato, a topic that warrants further experimentation. Rapid production of reactive oxygen species by plants is a hallmark of pathogen recognition and correlates with the HR. Working with disks cut from potato tubers that carry the *R1* late blight resistance gene, Doke [32] demonstrated that incompatible but not compatible races of *P. infestans* triggered production of reactive oxygen species. In our study, a gene (PGSC0003DMG400024754) encoding respiratory burst oxidase homolog protein B (NADPH oxidase RBOHB) was induced (based on between treatment comparisons) 6.6 fold in the *+RB* line but not in the WT line 48 hours after *P. infestans-*inoculation. This gene is known to be involved in the massive phase II oxidative burst induced in potato by pathogen infection [39] and offers further support that *RB-*mediated resistance to tuber late blight likely entails an HR or HR-like phenomenon.

Interestingly, HR is commonly associated with SA (salicylic acid) mediated responses. But our ontology bin analysis didn’t reveal SA metabolism as a differentiating factor for the tuber blight resistant and susceptible lines. Instead, our study reveals that the ET (ethylene) bin is predominantly associated with the incompatible interaction. Leon-Reyes et al. [40] reported that the antagonistic relationship between SA and JA [41] only exists when there is no strong production of ET. Nunez-Pastrana et al. [42] reported that ethylene but neither SA nor Methyl JA (jasmonic acid) induces resistance response against *Phytophthora capsici* in pepper. Extensive hormonal crosstalk [43] during plant responses to pathogens is a topic of extensive ongoing research and future results will likely yield new insights that will allow fine-tuning of our understanding of potato tuber-pathogen interactions and organ-specific defense responses in plants.

## Conclusion

We presented the first RNA-seq (or transcriptome dynamics) study focused on potato tuber responses to pathogen attack. We mapped approximately 400 million RNA-seq reads onto the recently published potato reference genome sequence, documenting the utility of RNA-seq for biological study of tetraploid cultivated potato. We identified sets of DE genes that distinguish resistant and susceptible lines. Our data suggest that potent regulation of defense genes and gene groups or ontology bins (e.g., *HIR* and WRKY transcription factors) plays a role in *RB*-mediated tuber defense. In particular, faster and stronger expression of defense related genes correlates with enhanced tuber blight resistance in the *+RB* transgenic line. In agreement with Kamoun et al. [34] and Doke [32], our data suggest that HR is likely a general form of resistance against late blight even in the potato tuber, an organ that develops below ground.

## Methods

### Plant material and RNA preparation

The tuber late blight susceptible nontransformed ‘Russet Burbank’ (WT, provided by Dr. Carl Rosen, UMN) and SP2211, a tuber late blight resistant transformed ‘Russet Burbank’ line carrying the *RB* transgene (+*RB*[5]) were examined in this study. Tubers were produced under standard cultural practices at the University of Minnesota Sand Plain Research Farm (Becker, MN). Tubers of each line were harvested and held for three days at room temperature before storage for six weeks at 11–13 degrees Celsius. *P. infestans* US8 isolate US940480 [3] was maintained on Rye A medium [44]. Prior to inoculations, sporangia were harvested from plates by physical scraping into distilled water. Inoculum was adjusted to 6.75*10^4^ sporangia/ml. Prepared inoculum was incubated for 1 hour at 4 degrees Celsius and then at room temperature for 30 minutes. Six week old WT and +*RB* tubers were inoculated using a modified whole tuber assay [6], as detailed below.

For RNA extractions and RNA-Seq, six tubers of each genotype were randomly selected, washed with deionized water, and allowed to air dry at room temperature for 24 hours. Tubers were wounded [0.2 cm*0.3 cm (depth*diameter)] at six sites spaced uniformly (~3 cm apart) across the tuber surface. Each wound was inoculated with 10 μl sporangial suspension (3 tubers per genotype) or water (mock treatment; 3 tubers per genotype). Inoculated tubers were stored in tightly sealed dark boxes under high (~95%) humidity and at room temperature for 72 hours (during which time all RNA-seq samples were collected as specified below) and then moved to 11–13 Celsius for disease development and phenotyping at 11 days post inoculation. From each of three replicate tubers per genotype, tuber tissue (0.7 cm*(0.5-0.8)cm) was collected using a cork borer from an inoculation site at time point 0 (pre-inoculation) and at 6, 12, 24, and 48 hours post inoculation (hpi). The sampled tissue includes cells from the periderm, cortex and medulla layers. The sixth inoculation site on each tuber was left intact and the tubers were phenotyped for late blight disease development 11 days later by multiplying measured length, width, and depth of disease lesions. Collected tissue samples (one tuber core per replicate tuber for each time point) were immediately frozen in liquid nitrogen and stored at −80 degrees Celsius. To reduce possible experimental variation, tissues collected for RNA-seq originated from a common experiment involving a single *P. infestans* inoculum preparation or water. In this study, each tuber is considered a biological replicate of the corresponding genotype by treatment combination. In total, 36 tissue samples from the two plant genotypes (WT and +*RB*) x three time points (0, 24, and 48 hpi) x two inocula (*P. infestans* or water) x three replicates were employed for RNA extraction. [Note: We collected tissue at 0, 6, 12, 24, and 48 hpi, but only 0, 24, and 48 hpi samples were subjected to RNA-seq]. Total RNA was extracted from frozen tissue using the SV Total RNA Isolation System (Promega Corporation, Madison, WI) according to manufacturer’s instructions. The quantity and quality of RNA samples were assessed using a Nanodrop 1000 machine (Thermo Fisher Scientific Inc., Wilmington, DE). High quality total RNA (5ug, 100 ng/μl) samples were sent to the University of Minnesota BioMedical Genomics Center (BMGC) for RNA-seq library preparation using the TruSeq SBS Kit (50 Cycles) (Illumina Inc., San Diego, CA) and paired end sequencing using an Illumina Hi-Seq 2000 machine (Illumina).

A separate, additional set of tubers (nine for WT and eighteen for +*RB*) were simultaneously subjected to the whole tuber assay (using the same inoculum and protocol described above), but without tissue collection, for the purposes of disease phenotyping.

### RNA-seq reads mapping and DE genes clustering

RNA-seq reads were quality filtered using SolexaQA packages [45] with default parameters and a length filter of greater than 27 bp for both ends of each read pair. Sequence data have been submitted to the NCBI Sequence Read Archive [accession number SRP022916]. Quality filtered RNA-seq reads were analyzed using the “Tuxedo Suite” software packages [46]: Bowtie [47] v0.12.7, Tophat [48] v1.3.2, Cufflinks [49] v1.1.0 and a reference genome of potato [9]. We suppressed Tophat [48] from identifying novel junctions. A total of eleven pair-wise comparisons [between time (4), between line (3), between treatment (4)] were made using Cuffdiff of the Cufflinks software packages. Differentially expressed (DE) genes are those genes that showed significantly (FDR adjusted p-value <0.001) different transcript levels among comparisons. False discovery rate correction (FDR) was done using the Benjamini Hochberg method [50].

Quality filtered RNA-seq reads were also mapped to reference transcript sequences of *P. infestans*[10] and different classes (see Champouret et al. [22] for details of classification) of *ipiO* (potential cognate avr effector of *RB*) gene sequences. The mapping was done using Bowtie [47] v0.12.7. Cufflinks software suites were not used in this analysis as underlying normalization assumptions were not met. Potato gene expression fold change values were used to cluster and partition genes into groups using hierarchical clustering and the complete linkage method in Cluster 3.0 [51] and visualized in Treeview software [52] using adjusted pixel settings (threshold 1.0).

The UNIX command line options for Tophat and Cuffdiff were as follows: “tophat –G genes.gff –o sample.out --no-novel-juncs –r 100 genome.fasta read.left.fq read.right.fq; cuffdiff –N –u –O cuffdiff.out genes.gff 1.bam,2.bam,3.bam 4.bam,5.bam,6.bam”. The UNIX command line options for Bowtie were as follows: “Bowtie -a -v 2 --best –M 1 --fr ref_bowtie_build −1 read1.fq −2 read2.fq”. Custom perl scripts were used to parse the mapping/alignment results into tabular formats. R statistical software [53] was used to generate various plots.

### Functional assignment and MapMan analysis of potato genes

To assign potato genes into functional categories (bins), we adopted MapMan ontology [54]. The Mercator (Max Planck Institute, Germany, Dr. Mark Lohse, personal communication) annotation pipeline was used to assign potato genes into functional bins by searching a variety of reference databases. The mapping file was manually curated to remove suspicious assignments. Water- and *P. infestans*-inoculated samples were compared using Fisher’s exact test to identify ontology bins enriched with significantly up or down regulated DE (FDR <0.001) genes. WT and *+RB* lines were compared using the Wilcoxon rank-sum test to identify functional bins that behaved differently. Resulting p-values from Fisher’s exact tests or Wilcoxon rank-sum tests were subjected to multiple test correction using the Benjamini Hochberg method [50].

### Real-time quantitative RT-PCR

For determining the transcript levels of the transgene *RB*, we used a previously established qRT-PCR protocol [5,55]. For RNA-seq validation, Primer Express 3.0 (Applied Biosystems, Foster City, CA, USA) was used with default parameters to generate primer pairs for selected transcripts (Additional file 11). Total RNA (150 ng, 30 ng/ul) from diluted stocks of the same RNA that was subjected to RNA-seq was used in each reverse transcription reaction using the SuperScript III First Strand Synthesis Kit (Life Technologies Inc., Carlsband CA) according to manufacturer’s instructions. All qPCRs were performed using the Power SYBR Green Master Mix (Life Technologies) and an ABI 7500 Real Time System (Applied Biosystems). Target gene transcript levels were normalized to *EF1α*[56] using 2^-ΔCT^ values. Normalized gene expression levels were compared with RNA-seq FPKM values derived from Cufflinks.

## Competing interests

The authors declare that they have no competing interests.

## Authors’ contributions

LLG was responsible of all bench work and informatics analyses of RNA-seq reads filtering, mapping, DE genes detection, and ontology regulation analysis. ZJT provided some informatics support, especially in relation to a pilot RNA-seq project. BPM identified transgenic line SP2211 (+*RB*), pioneered tuber resistance phenotyping, and developed molecular assays for transgene transcription measurement. JMB provided the conceptual impetus for this work, suggestions on all aspects of experimental design and data analysis, and secured funding for this project. LLG wrote this manuscript and ZJT, BPM, and JMB provided comments and suggestions to improve the manuscript. All authors read and approved the final manuscript.

## Supplementary Material

Additional file 1 **RNA-seq FPKM and qPCR correlation.** First column is potato tuber sample/IDs. Remaining columns indicate qPCR results and RNA-seq FPKM values for each potato gene, as indicated. The per gene and average correlations between RNA-seq and qPCR are listed at the bottom of the page. Click here for file

Additional file 2 Fold change values for transcript levels of each of the 39,301 potato genes in each of the 20 comparisons. Click here for file

Additional file 3 **MA plots (hybridization intensity plotted against the fold change in expression) of between line comparisons.** Each x-axis indicates mean gene expression levels [log2(FPKM1)+log2(FPKM2)] across the two time points selected for comparison. Each y-axis indicates fold change values [log2(FPKM1/FPKM2)]. (A) WT compared to +*RB* at 0 hpi; (B): WT compared to +*RB* at 24 hpi; (C): WT compared to +*RB* at 48 hpi. Click here for file

Additional file 4 **Hierarchical clustering and Treeview visualization of 1,767 DE genes (determined based on between time point comparisons; Note: genes that are DE in between time point comparisons in water-inoculated samples were exclude from this analysis) show that DE gene regulation patterns in compatible and incompatible interactions are predominantly similar.** Each column represents a comparison between two time points. Column one: 0 hpi to 24 hpi in WT; column two: 0 hpi to 24 hpi in +*RB*; column three: 24 hpi to 48 hpi in WT; column four: 24 hpi to 48 hpi in +*RB*. Red indicates up-regulation, green indicates down regulation. Left panel: the overall pattern of the 1,767 genes. Right panel: Magnified images of small gene clusters. Click here for file

Additional file 5 **Transcription of WRKY genes is highly induced in *+RB* but not WT.** Column one and two are gene ID and PGSC annotation descriptions. Columns three to six are log2 fold change values derived from between treatment comparisons (*P. infestans-* vs. water-inoculated). Values highlighted in red are statistically different between the *+RB* and WT lines. Click here for file

Additional file 6 **Tubers of ‘Russet Burbank’ (WT) and SP2211 (*+RB*) were inoculated with *P. infestans* and water.** We compared the RNA-seq FPKM counts for WT and +*RB* using all 39,031 gene models included in the Potato Genome Sequencing Consortium (PGSC) v3 dataset (i.e., all genes were included, regardless of whether or not a given gene was DE). Genes were grouped into ontology bins using a Mapman mapping file. Each column represents a comparison between the two genotypes at a defined time point post inoculation, as indicated. Bins in blue are transcribed at higher levels in WT than in *+RB;* bins in red are transcribed at higher levels in *+RB* than in WT; bins in white did not significantly differ in transcript levels between WT and *+RB.* Results indicate that stronger activation of defense bins, including stress responses and receptor kinases, occurred in +*RB* (the tuber blight resistant line). Click here for file

Additional file 7 **In a pilot study, WT and +*RB* samples at 0, 24 and 48 hours post *P. infestans* inoculation were collected from three bio-reps and pooled into a single composite sample for RNA-seq.** A total of 146.6 million single end Illumina reads (51 bp) were filtered and mapped to the reference potato genome using SolexaQA and Tuxedo software suite packages [9]. Cuffdiff was used to generate log2 transformed fold change values for each between genotype comparisons at 0, 24 and 48 hpi. Mapman analyses and Wilcoxon rank sum tests were performed (see methods). Columns one, two, and three represent +*RB* vs. WT comparisons at 0, 24 and 48 hpi, respectively. Blue bins show higher transcription in WT; red bins show higher transcription in +*RB*. Note that +*RB* has faster and stronger activation of defense related bins (stress and receptor kinases) at 24 and 48 hpi. Click here for file

Additional file 8 **Log2 transformed total read counts that mapped to *Phytophthora infestans* transcripts.** The X-axis indicates different time points (0, 24, 48 hpi) post *P. infestans* inoculation. The Y-axis indicates log2 transformed total mapped reads count. Results indicate an increase in *P. infestans* RNA-seq reads in the WT but not the +*RB* line over time. Click here for file

Additional file 9 **Bowtie RNA-seq read mapping results for 4,634 *Phytophthora infestans* genes.** The first column contains gene ID, the second column indicates whether the gene is a predicted RxLR effector gene, each remaining column contains mapping summary results for each of the 36 potato tuber samples. Click here for file

Additional file 10 **Bowtie RNA-seq reads mapping results for *ipiO* genes.** The first and second columns are ref id and short IDs for the *ipiO* genes adopted from Champouret et al. [22]. Each remaining column contains a bowtie mapping summary for each sample following tuber inoculation with *Phytophthora infestans.* Click here for file

Additional file 11 qRT-PCR primer sequences for 20 selected potato genes. Click here for file
