# Supplementary figures and images for: Insights into organ-specific pathogen defense responses in plants: RNA-seq analysis of potato tuber-Phytophthora infestans interactions
Source: BMC Genomics. 2013 May 23;14:340. doi: 10.1186/1471-2164-14-340 (PMC3674932; doi:10.1186/1471-2164-14-340)

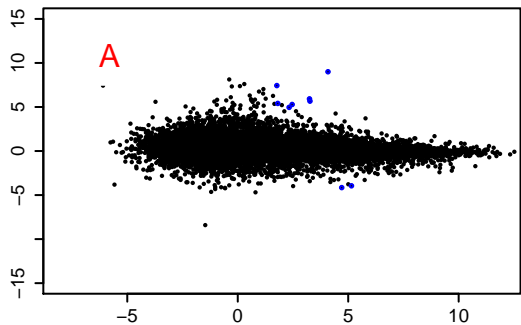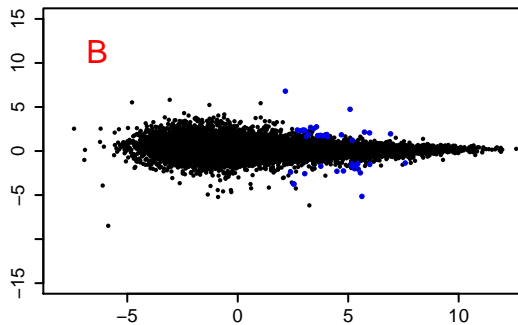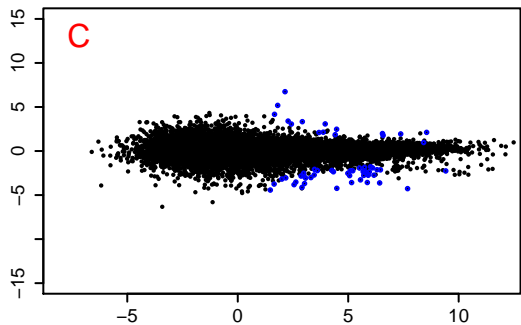

Supplement: Additional file 3 — MA plots (hybridization intensity plotted against the fold change in expression) of between line comparisons. Each x-axis indicates mean gene expression levels [log2(FPKM1)+log2(FPKM2)] across the two time points selected for comparison. Each y-axis indicates fold change values [log2(FPKM1/FPKM2)]. (A) WT compared to +RB at 0 hpi; (B): WT compared to +RB at 24 hpi; (C): WT compared to +RB at 48 hpi. [file 1471-2164-14-340-S3.pdf]

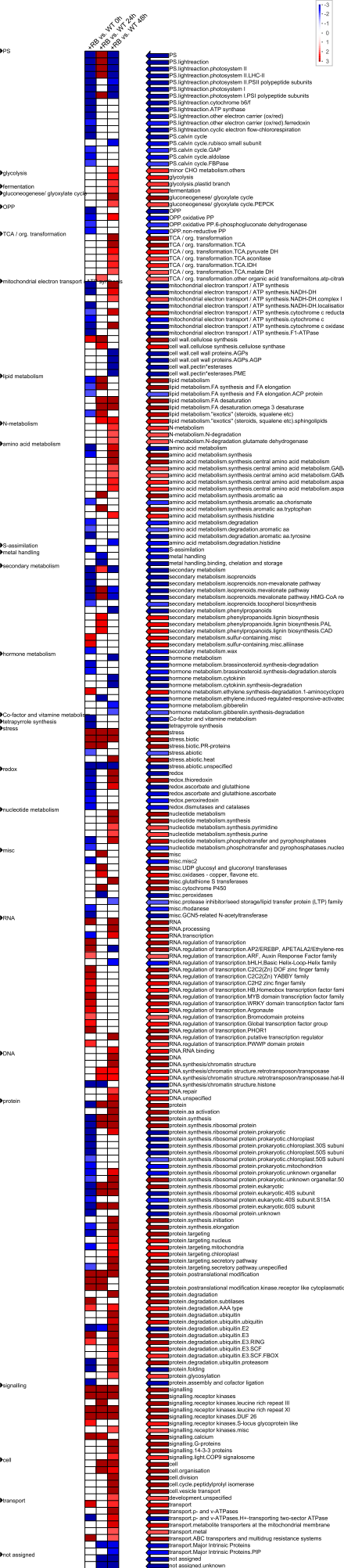

Supplement: Additional file 6 — Tubers of ‘Russet Burbank’ (WT) and SP2211 (+RB) were inoculated with P. infestans and water. We compared the RNA-seq FPKM counts for WT and +RB using all 39,031 gene models included in the Potato Genome Sequencing Consortium (PGSC) v3 dataset (i.e., all genes were included, regardless of whether or not a given gene was DE). Genes were grouped into ontology bins using a Mapman mapping file. Each column represents a comparison between the two genotypes at a defined time point post inoculation, as indicated. Bins in blue are transcribed at higher levels in WT than in +RB; bins in red are transcribed at higher levels in +RB than in WT; bins in white did not significantly differ in transcript levels between WT and +RB. Results indicate that stronger activation of defense bins, including stress responses and receptor kinases, occurred in +RB (the tuber blight resistant line). [file 1471-2164-14-340-S6.pdf]

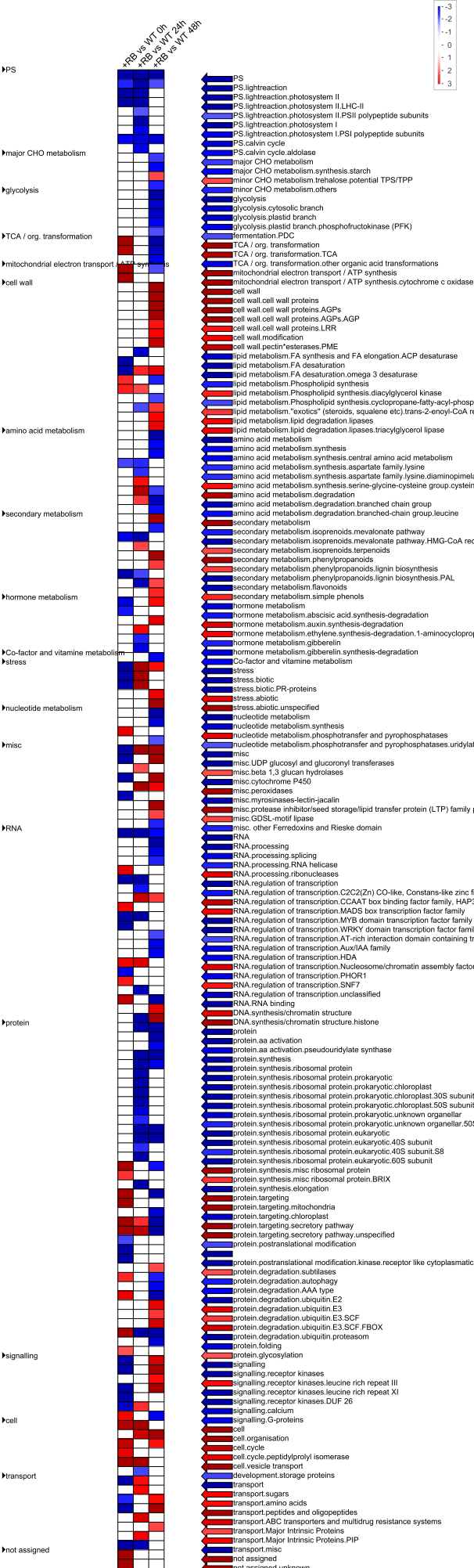

Supplement: Additional file 7 — In a pilot study, WT and +RB samples at 0, 24 and 48 hours post P. infestans inoculation were collected from three bio-reps and pooled into a single composite sample for RNA-seq. A total of 146.6 million single end Illumina reads (51 bp) were filtered and mapped to the reference potato genome using SolexaQA and Tuxedo software suite packages [9]. Cuffdiff was used to generate log2 transformed fold change values for each between genotype comparisons at 0, 24 and 48 hpi. Mapman analyses and Wilcoxon rank sum tests were performed (see methods). Columns one, two, and three represent +RB vs. WT comparisons at 0, 24 and 48 hpi, respectively. Blue bins show higher transcription in WT; red bins show higher transcription in +RB. Note that +RB has faster and stronger activation of defense related bins (stress and receptor kinases) at 24 and 48 hpi. [file 1471-2164-14-340-S7.pdf]

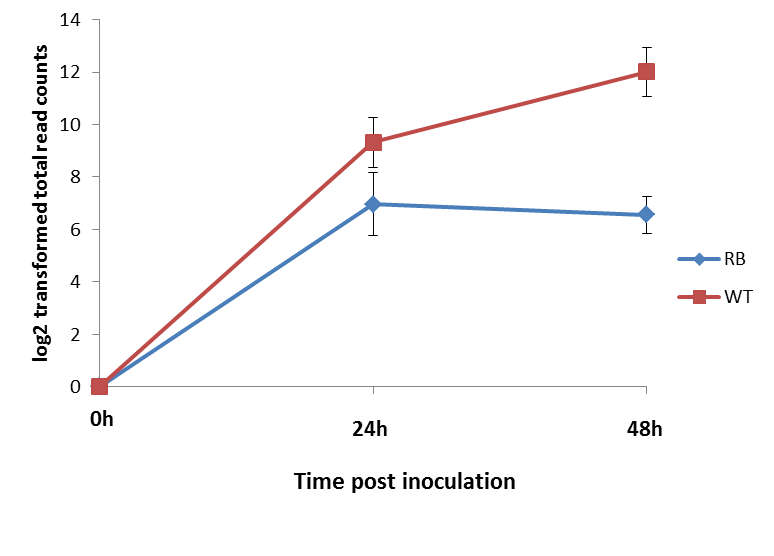

Supplement: Additional file 8 — Log2 transformed total read counts that mapped to Phytophthora infestans transcripts. The X-axis indicates different time points (0, 24, 48 hpi) post P. infestans inoculation. The Y-axis indicates log2 transformed total mapped reads count. Results indicate an increase in P. infestans RNA-seq reads in the WT but not the +RB line over time. [file 1471-2164-14-340-S8.png]
