# Supplementary material for: Insights into organ-specific pathogen defense responses in plants: RNA-seq analysis of potato tuber-Phytophthora infestans interactions
Source: BMC Genomics. 2013 May 23;14:340. doi: 10.1186/1471-2164-14-340 (PMC3674932; doi:10.1186/1471-2164-14-340)
Supplement: Additional file 4 — Hierarchical clustering and Treeview visualization of 1,767 DE genes (determined based on between time point comparisons; Note: genes that are DE in between time point comparisons in water-inoculated samples were exclude from this analysis) show that DE gene regulation patterns in compatible and incompatible interactions are predominantly similar. Each column represents a comparison between two time points. Column one: 0 hpi to 24 hpi in WT; column two: 0 hpi to 24 hpi in +RB; column three: 24 hpi to 48 hpi in WT; column four: 24 hpi to 48 hpi in +RB. Red indicates up-regulation, green indicates down regulation. Left panel: the overall pattern of the 1,767 genes. Right panel: Magnified images of small gene clusters. [file 1471-2164-14-340-S4.pdf]

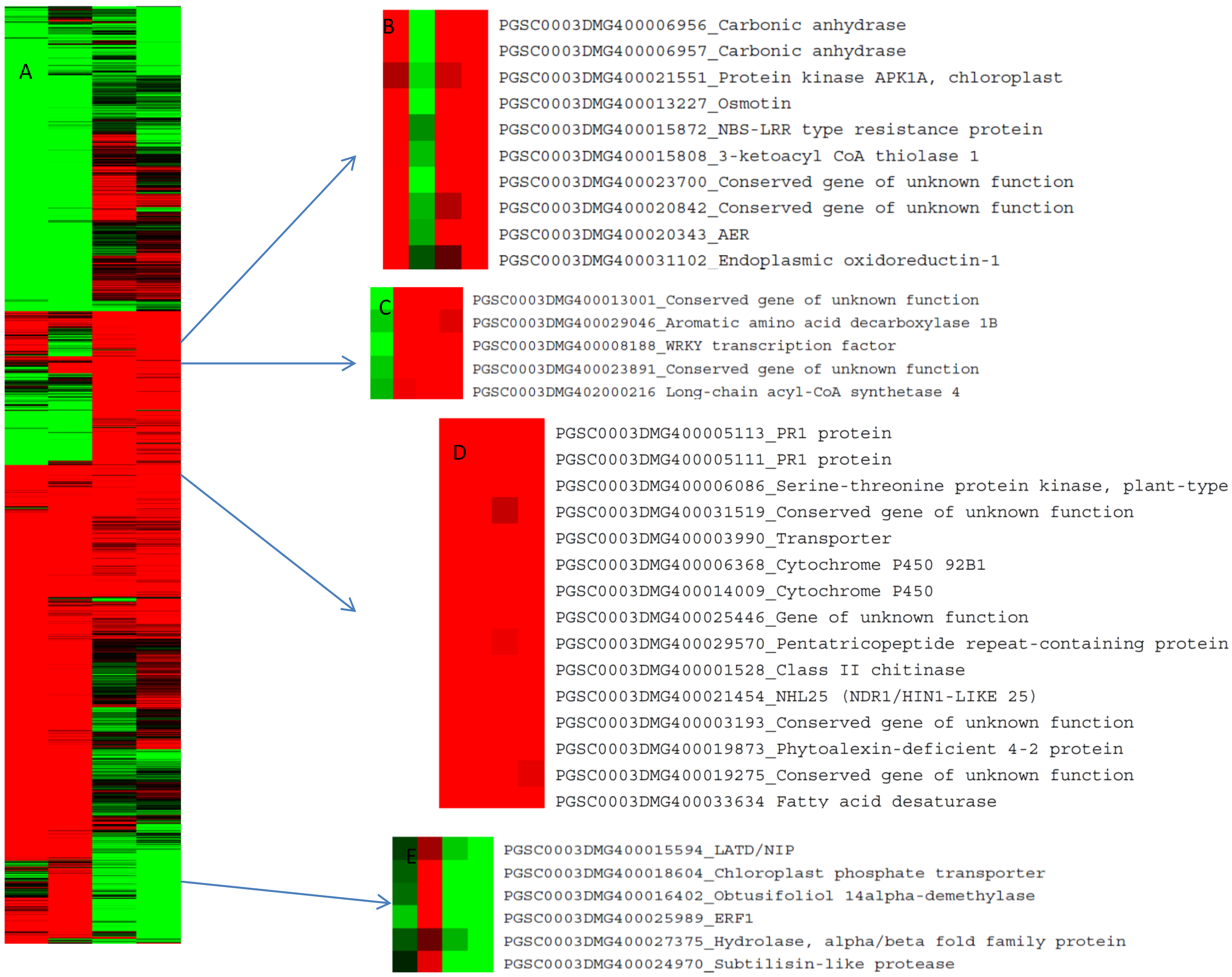
A
B
PGSC0003DMG400006956_Carbonic anhydrase
PGSC0003DMG400006957_Carbonic anhydrase
PGSC0003DMG400021551_Protein kinase APK1A, chloroplast
PGSC0003DMG400013227_Osmotin
PGSC0003DMG400015872_NBS-LRR type resistance protein
PGSC0003DMG400015808_3-ketoacyl CoA thiolase 1
PGSC0003DMG400023700_Conserved gene of unknown function
PGSC0003DMG400020842_Conserved gene of unknown function
PGSC0003DMG400020343_AER
PGSC0003DMG400031102_Endoplasmic oxidoreductin-1
C
PGSC0003DMG400013001_Conserved gene of unknown function
PGSC0003DMG400029046_Aromatic amino acid decarboxylase 1B
PGSC0003DMG400008188_WRKY transcription factor
PGSC0003DMG400023891_Conserved gene of unknown function
PGSC0003DMG402000216 Long-chain acyl-CoA synthetase 4
D
PGSC0003DMG400005113_PR1 protein
PGSC0003DMG400005111_PR1 protein
PGSC0003DMG400006086_Serine-threonine protein kinase, plant-type
PGSC0003DMG400031519_Conserved gene of unknown function
PGSC0003DMG400003990_Transporter
PGSC0003DMG400006368_Cytochrome P450 92B1
PGSC0003DMG400014009_Cytochrome P450
PGSC0003DMG400025446_Gene of unknown function
PGSC0003DMG400029570_Pentatricopeptide repeat-containing protein
PGSC0003DMG400001528_Class II chitinase
PGSC0003DMG400021454_NHL25 (NDR1/HIN1-LIKE 25)
PGSC0003DMG400003193_Conserved gene of unknown function
PGSC0003DMG400019873_Phytoalexin-deficient 4-2 protein
PGSC0003DMG400019275_Conserved gene of unknown function
PGSC0003DMG400033634 Fatty acid desaturase
E
PGSC0003DMG400015594_LATD/NIP
PGSC0003DMG400018604_Chloroplast phosphate transporter
PGSC0003DMG400016402_Obtusifoliol 14alpha-demethylase
PGSC0003DMG400025989_ERF1
PGSC0003DMG400027375_Hydrolase, alpha/beta fold family protein
PGSC0003DMG400024970_Subtilisin-like protease
