# Supplementary material for: Insights into organ-specific pathogen defense responses in plants: RNA-seq analysis of potato tuber-Phytophthora infestans interactions
Source: BMC Genomics. 2013 May 23;14:340. doi: 10.1186/1471-2164-14-340 (PMC3674932; doi:10.1186/1471-2164-14-340)
Supplement: Additional file 5 — Transcription of WRKY genes is highly induced in +RB but not WT. Column one and two are gene ID and PGSC annotation descriptions. Columns three to six are log2 fold change values derived from between treatment comparisons (P. infestans- vs. water-inoculated). Values highlighted in red are statistically different between the +RB and WT lines. [file 1471-2164-14-340-S5.pdf]

Additional file 6 WRKY genes highly induced in +RB but not WT

log2 expression fold values (red highlighted are values that are statistically significant)

Gene	PGSC.annot	WT_24h	+RB_24h	WT_48h	+RB_48h
PGSC0003DMG400000211	WRKY transcription factor	0.361932	-0.10115	1.3649	2.76934
PGSC0003DMG400005835	WRKY transcription factor-30	-0.24461	0.227315	2.54673	3.62647
PGSC0003DMG400008188	WRKY transcription factor	-0.60055	-0.4471	2.92134	5.6014
PGSC0003DMG400011633	WRKY-type transcription factor	0.592792	0.604168	0.692155	2.70031
PGSC0003DMG400016441	WRKY protein	0.763239	0.177539	0.007094	1.09009
PGSC0003DMG400016769	Double WRKY type transfactor	0.098889	-0.23761	0.283502	1.98727
PGSC0003DMG400019824	JA-induced WRKY protein	-0.63359	-1.14316	0.855998	3.55835
PGSC0003DMG400020206	WRKY transcription factor-b	1.52349	0.511368	2.55535	4.27083
PGSC0003DMG400020608	DNA-binding protein 3	0.908722	-0.45846	1.61096	4.33967
PGSC0003DMG400021895	WRKY-type DNA binding protein	0.182197	-1.13779	0.576008	1.97609
PGSC0003DMG400028520	WRKY transcription factor 1	-0.26909	-0.6326	0.270862	1.58399
PGSC0003DMG400031140	WRKY transcription factor	0.383975	-3.23026	2.4424	6.9517
PGSC0003DMG401010558	WRKY-A1244	0.056465	-0.15241	-0.95852	-2.05383
PGSC0003DMG402007388	MRNA, 1346 bp sequence	2.87749	-10	3.13664	5.30573
